# Information content and acoustic structure of male African elephant social rumbles

**DOI:** 10.1038/srep27585

**Published:** 2016-06-08

**Authors:** Angela S. Stoeger, Anton Baotic

**Affiliations:** 1 Mammal Communication Lab, Department of Cognitive Biology, University of Vienna, Vienna, 1090, Austria

## Abstract

Until recently, the prevailing theory about male African elephants (*Loxodonta africana*) was that, once adult and sexually mature, males are solitary and targeted only at finding estrous females. While this is true during the state of ‘musth’ (a condition characterized by aggressive behavior and elevated androgen levels), ‘non-musth’ males exhibit a social system seemingly based on companionship, dominance and established hierarchies. Research on elephant vocal communication has so far focused on females, and very little is known about the acoustic structure and the information content of male vocalizations. Using the source and filter theory approach, we analyzed social rumbles of 10 male African elephants. Our results reveal that male rumbles encode information about individuality and maturity (age and size), with formant frequencies and absolute fundamental frequency values having the most informative power. This first comprehensive study on male elephant vocalizations gives important indications on their potential functional relevance for male-male and male-female communication. Our results suggest that, similar to the highly social females, future research on male elephant vocal behavior will reveal a complex communication system in which social knowledge, companionship, hierarchy, reproductive competition and the need to communicate over long distances play key roles.

Determining the information content of vocalizations is a curial step towards understanding a species’ communication system, but the structure and function of specific call types may differ with age, gender, or reproductive state. In recent decades, research on African elephant vocal communication strongly focused on the acoustic structure and function of female elephant rumbles, low-frequency and harmonically rich vocalizations used in long- and short-distance contexts^1^,^2^,^3^,^4^. Other studies investigated female social knowledge and networks of vocal recognition^5^,^6^ and, more recently, female auditory discrimination skills^7^,^8^. While some information is available about infant, calf and juvenile vocalizations^9^,^10^, male elephant vocal communication has received considerably less research attention.

Recent studies on male African elephants investigated social dynamics and male-male relationships, highlighting that they are not as asocial as previously thought^11^,^12^,^13^,^14^,^15^. Males start to leave their natal family at an average age of 14 years, but independence is a gradual and lengthy process^16^,^17^. African elephants engage in contest polygyny, with females coming into estrus for 4–6 days once every four years^18^. Male elephant society is generally regulated by dominance and a reproductive state termed musth^12^,^19^, a temporary condition in adult bulls characterized by increased aggressive behavior and elevated androgen levels^20^,^21^. Although musth is not an explicit requirement for male reproductive success, paternity analyses have revealed that approximately 75–80% of offspring can be attributed to musth males^22^,^23^. Whereas musth-bulls spend much time around female groups, non-musth males often spend their time in geographically distinct bull areas and do tolerate the company of other males (non age-mates and age-mates)^12^,^19^. Social networks are larger and denser when not in musth^15^, and all-male coalitions and companionship have been reported^12^. Nonetheless, the nature of these relationships (short- or long-term) remains unclear. Although musth is considered as ‘the competitive state’ in bull elephants^21^, it is suggested that competitor assessment takes place during non*-*musth periods as well, possibly by reaffirming dominance hierarchies or renegotiating relations based on dynamic variables such as body condition^15^.

Vocalizations in animals often encode important information about the vocalizer’s phenotype such as body size^24^,^25^,^26^. In some species, vocalizations are used to determine the potential outcome of agonistic or hierarchical contests between males and are an important component of intrasexual competition^27^,^28^,^29^. Here, we analyze the acoustic structure and the information content of rumbles of non-musth male African elephants recorded in various social contexts. Since social status, body size and age are important correlates of reproductive success^22^, and elephant rumbles are classical long-distance signals, male-male competition is likely to be related to the acoustic structure of male elephant vocal signals in general.

Species-specific and individual mechanisms of sound production determine the characteristics of the vocal output. The production mechanism of the elephant *rumble* follows the principles of the source and filter theory of human speech production^2^,^3^,^30^,^31^. The sound waves are generated by passive vocal fold vibration in the larynx (*the sound source*). The fundamental frequency, mainly determined by the mass of these vibrating vocal folds^32^, is an important acoustic feature also in elephants. It generally decreases with age^33^, which is a maturational effect, and has been shown to differ according to context and motivational state in adult female^34^,^35^,^36^,^37^,^38^ and infant African elephants^9^,^10^. Following sound generation, the sound wave travels through the supra-laryngeal vocal tract. The vocal tract acts like a *filter* on the sound spectrum, selectively amplifying and attenuating certain frequencies (formants) because of resonances within the oral and nasal cavities^30^,^32^. Formants are generally determined by the length and shape of the vocal tract, with longer tracts producing lower, more closely spaced formants^30^. Due to this strong correlation, formants are suggested to serve as a honest cue to body size in many (probably most) vertebrates^26^. Morphological adaptations (from a proximate and evolutionary (ultimate) view) to elongate the vocal tract in order to lower formants have been reported in several mammalian species (most often in males), with the size exaggeration hypothesis^39^ being proposed to justify most of these observations^40^,^41^,^42^.

Elephants have an extremely elongated nasal vocal tract (proboscis) and use it in rumble production^2^,^3^. Female elephants have been shown to produce rumbles orally and nasally with considerable variation in the resulting formant frequencies^35^. The values of the first two formants reflect the estimated lengths of the vocal paths, corresponding to a vocal tract length (VTL) of around 2 meters for nasal and 0.7 meters for oral rumbles in the investigated female individuals^35^. Further studies on female elephant rumbles have documented other formant variations depending on context and state of arousal. Specifically, an upward shift in the second formant seems to alert other elephants to potential danger^36^, and dominant females engaged in hierarchical interactions produce rumbles with particularly low formant dispersion^37^.

During the period of musth, males emit a structurally distinct musth-rumble suggested to advertise the hormonal state to both females and other males^1^,^20^. Basic acoustic analyses have been done, and musth rumbles are described as being pulsated, with a fundamental frequency of 11 to 17 Hz and a mean duration of about 4.4 seconds^1^,^34^,^43^. Outside the context of musth, next to nothing is known about male elephant vocal signals.

The aim of the current study was to determine whether male African elephant rumbles have the potential to convey information about maturity and individuality based on source and filter theory. This approach provides the first important indications on their potential functional relevance for male-male and male-female communication apart from the context of musth.

## Results

### Acoustic cues to maturity

**Figure 1:**
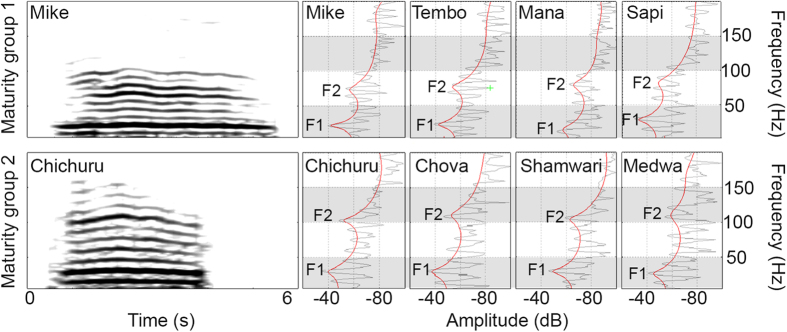
Spectrograms and spectra of individual rumbles presenting structural differences between the two maturity groups. The formants (F1 and most obviously F2) are lower in the rumbles of maturity group 1 compared to those of maturity group 2. Spectrograms (Hamming window, bandwidths = 2 Hz, 75% overlap) and spectra were generated in S_Tools Stx.

We used permuted discriminant function analysis (pDFA) to examine differences in rumbles according to maturity (age and size) of ten male African elephants that were categorized into two maturity groups (**1**) males with shoulder height above 3 meters and over 25 years (n_ind_ = 5, n_calls_ = 79), and (**2**) males with shoulder height below 3 meters and younger than 25 years (n_ind_ = 5, n_calls_ = 83) (Table 1). The initial ANOVA revealed no significant difference in most of the shape- and temporal-related parameters of the fundamental frequency (‘frequency variability’, ‘inflection factor’, ‘minimum frequency location’ as well as ‘start, middle and final slope’, ‘time min to max’, Table 2), thus they have been excluded from analysis. The subsequent principal component analysis (PCA) reduced the remaining parameter to 4 factors explaining 82.4% of the variation (see Supplemental Table S1). The pDFA (entering maturity group as test factor and individuality as control factor) resulted in 98% correct classification applying cross-validation (94% error reduction), demonstrating that maturity groups in male elephants were clearly discriminable based on acoustic features of rumble vocalizations (p = 0.003). The main discriminative factors were formant frequencies (formant 1 and formant 2) as well as absolute fundamental frequency values (Fig. 1, Table 3).

Most of our elephant rumble recordings possessed two measurable formant frequencies (not uncommon for elephants^2^,^7^, but see Supplementary Figs S1–S3, for rumble examples with three and four formants). Based on formant location and dispersion (of formant 1 and formant 2), the estimated mean vocal tract lengths in meters ± SD for the analyzed rumbles of the males of maturity group 1 was 3.21 m ± 0.51, for maturity group 2 it was 2.36 m ± 0.26.

### Acoustic cues to individual identity

We applied pDFA in order to investigate the extent of individuality in male African elephant rumbles. Since the comparison of maturity groups revealed that maturity significantly impacts the acoustic structure, we controlled our analysis for this particular factor. The potential of individual coding (PIC) was <1 for the slope features ‘frequency variability’, ‘peak by mean’, and ‘minimum frequency location’ (Table 2), and these have therefore been excluded from the subsequent analysis. The remaining variables were entered into PCA and reduced to 6 factors, explaining 81.6% of variation (see Supplemental Table S2). Entering all 6 PCA factors as variables, individuality as test factor and maturity group as restriction factor, the pDFA correctly classified 55% applying cross-validation method (44% error reduction). The significance level of p = 0.001 shows that the individual males were evidently discriminated based on acoustic features.

## Discussion

This paper presents the first comprehensive acoustic analysis of male elephant vocalizations, considering source- and filter-related acoustic features of low-frequency social rumbles. Our results demonstrate that male elephant rumbles, from a structural point of view, encode information on the physical attributes of the caller, most prominently information about maturity. Reproductive success in male African elephants is positively correlated to size and age^22^, and the driving force of being dominant to successfully reproduce seems reflected in the structure of male elephant vocalizations, as has been shown in several other mammalian species^41^,^42^. Although we analyzed rumbles of males living under human care, the information on a caller’s phenotype is not expected to vary between captive and wild elephants. This is because the vocal characteristics are determined by individual- and species-specific mechanisms of sound production, which are independent of living conditions.

Male elephants grow throughout their lives; a 20-year-old bull weighs about 3500 kg (approximately the weight of a fully grown female), whereas a 40-year-old male weighs between 6000 and 7000 kg^44^. In captivity, males successfully reproduce and get into musth well below the age of 20, when male-male competition does not occur^45^,^46^ (since zoos usually only keep one male). In the wild, maturity is a decisive factor, and younger males are naturally suppressed by the physical presence of older ones. A discrimination between rumbles of the two maturity groups and differences in formant values were therefore expected, but the high classification success achieved was still surprising. This supports the hypothesis that formants serve as a honest cue to body size in most vertebrates^26^, including the biggest terrestrial mammals. The fundamental frequency was further significantly lower in rumbles of maturity group 1, with the lowest measured values being well below 10 Hz. In comparison, the average fundamental frequency of adult female rumbles in similar low-arousal contexts ranges between 14 and 18 Hz^34^.

Acoustic cues to maturity might also be relevant for intersexual communication, although not much information is available on female choice in elephants. Estrus females, however, seem to prefer males of higher age/size classes as mates^47^. Moss^47^ observed significantly more successful chases of females by males of older age classes, compared to younger bulls. Female mate choice can significantly drive the evolution of male vocalizations^48^. Females across several species use acoustic indicators to body size or strength to assess potential mates^49^,^50^,^51^,^52^. Whether this is also the case in elephants remains to be investigated, and future research should focus on the role of vocalizations as well as general mechanisms of mate choice in more detail.

The estimated vocal tract length of the elephants whose vocalizations were analyzed strongly indicates that the rumbles were nasally emitted. This result was expected because the rumbles were preselected for analysis to be associated with low-arousal social contexts, and elephants apparently produce oral rumbles mainly in high-arousal situations^35^. Nonetheless, further investigations of these two rumble production types, particularly in males, are necessary. The estimated average vocal tract length of about 3.21 meters in large males is reasonable considering their shoulder height (well above 3 meters) and the nasal path^31^. This is the longest so far reported in terrestrial mammals. The estimated values for the younger bulls of maturity group 2 resemble those of adult females^2^,^3^.

Dominant females engaged in hierarchical interactions produce rumbles with particularly low formant dispersion, which indicates that lowering formants signals physical dominance to competitors^37^. In humans, male dominance competition has been proposed as the main evolutionary force behind men’s low voice^53^. Unfortunately, our data have not allowed identifying formant variations due to differences in social rank or during hierarchical interactions yet, but they indicate that social structure and group composition (e.g. males of the same age) might be related to formant structure in males of similar size. We will investigate these aspects in male elephant vocalizations in the future. Certain structures of the elephant vocal tract (e.g. the hyoid, the pharyngeal pouch, the trunk per se) are highly flexible^54^. Accordingly, elephants seem capable (both, from an morphological and cognitive point of view)^55^ of producing a diversity of meaningful formant variations that remain to be described and functionally understood. Although, it has been shown that elephants do respond distinctively to vocalizations that differ in acoustic parameter including formant structure^7^, playback experiments need to be done to verify perception of format frequencies and formant variation in both, male and female individuals.

Our results further reveal cues to individuality in male elephant rumbles. We achieved similar classification results as reported in studies on individuality in female rumbles (considering the cross-validated results)^2^,^56^. While formants were still distinctive, absolute frequency values as well as shape-related features of the fundamental frequency differed between individuals. This is similar to the discriminative parameters found in female rumbles^2^,^56^. Widespread benefits might be associated with vocal distinctiveness of male elephants. Individual distinctiveness and recognition (although acoustic discrimination abilities still need to be tested in male elephants) might be used to discriminate a mate, offspring, sibling, social affiliate or rival. In female elephants, individual recognition has been shown to be particularly relevant for socially affiliated individuals^2^,^5^. Such an advantage, and thus selection for signalers to be memorably different, might provide mechanisms that increase phenotypic variability^57^. A better understanding of these mechanisms would require investigating intra- (concerning males) and intersexual vocal recognition among elephants. Females have been shown to distinguish the calls of family and bond-group members from those of females outside of these categories; individuals have to be familiar with an estimated minimum of 100 adult female calls^5^, and males maybe have a similar distinctive knowledge of other males associated with social affiliation and rank (which would be particularly interesting for males of similar size and strength). Intersexual recognition could be similarly important. Estrus females, for example, might prefer companionship of familiar (though not related) over unfamiliar males, but as mentioned above, female choice in elephants is relatively unknown.

Our study focused on male social rumbles, but musth rumbles are equally interesting. This is because they transmit the hormonal state in addition to the other physiological traits, calling for investigating these special and distinctive rumbles in more detail. With regard to overall vocal behavior, male African elephants are generally less vocal than females because they do not have to vocally coordinate the movement of a herd or summon calves. Notwithstanding, social knowledge, companionship, hierarchy, reproductive competition and the need to communicate over long distances are some of the aspects that drive male elephant behavior and shaped exceptionally powerful and impressive (even for human auditors) vocalizations. Therefore, although taciturn compared to females, closer examination reveals that male elephants are ‘men of their word’: if they vocalize, it is worth listening.

## Methods

### Ethic statement

This study complies with all applicable Austrian and South African laws and was conducted in accordance with the Guidelines for the Treatment of Animals in Behavioral Research and Teaching^58^. The owners of the elephants issued permission for the research to be conducted by the authors. The elephants were recorded without performing any manipulations and without conducting playback experiments. Research was only observant and did not affect the housing, the daily routine, the behaviors, diet or management of the animals. Therefore, no ethics committee approval was required.

### Study subjects and housing

The subjects in this study were 13 male elephants (non-musth) aged between 18 and 33 years located at privately owned elephant keeping institutions in South Africa (Table 1). The males have social contact to other males and females during the day and spend the night in separate stables next to each other (but again, do have tactile, visual and acoustic contact with the rest of their herd). All elephants were fully habituated to human presence and at daytime free to roam around in areas of about 300 to 4500 ha. For investigating acoustic cues to maturity and individuality we recorded male elephants in controlled conditions to ensure multiple high quality samples per individual of known age and size.

Independent of this research, all individuals receive GnRH vaccination^59^ approximately every 5 months since several years prior to this study in order to prevent the males from entering the state of musth. Overall median fecal androgen metabolite levels (2.37 μg/g DW) seem to be comparable with fAM concentrations revealed for free-ranging adult male African elephants, when no physical signs of musth are present (2.13 μg/g)^60^.

### Data collection

Recording sessions were conducted throughout the day between 7 a.m. and 5 p.m. The broad contexts of the recording situations were vocalizations and social interactions during browsing. We followed the elephants by foot accompanied by elephant handlers for security reasons, did not interact with the animals and passively recorded and observed at distances ranging from 10 to 50 meter. Caller identification works best during calm contexts such as browsing, where individuals divide up and decentralize and when the focus elephants are close and the observer can perceive the sound; e.g. observer <20 m from the elephant. In order to allocate vocalizations to individuals, we usually focused on particular individuals for a certain time period. We observed the focus elephant and noted the ID only if both authors agreed upon the calling individual. In addition to auditory cues, we considered optical cues such as lifted or spread ears, and general body postures and changes of posture to identify the vocalizing individual. We did also use video recordings to verify vocalizing individuals during data annotation.

We documented and recorded all produced vocalizations that could be individually allocated. We used an omni-directional Neumann microphone (KM 183) modified for recording frequencies below 20 Hz (flat recording down to 5 Hz) connected to a 722 Sound Device HDD recorder at 48 kHz sampling rate. Concomitant video recordings were done using a Sony DHC-SD909 camcorder in HD quality. This helped to verify field notes later in the laboratory during data annotation.

Shoulder height was measured by the elephant handlers using a telescopic meter (Telefix 4 meter) with accuracy of a few centimeters. Slight irregularities in the terrain or the standing position/shift of the elephants can affect the absolute measures.

Dung samples were collected during our stay at each institution in order to get a broad idea of fecal androgen metabolite levels of the individuals during the recording period. We did not correlate dung samples with recording sessions. Approximately 100–250 g of feces was taken from the middle of a dung bolus shortly after an animal had defecated and moved away. The samples were stored at −20 °C at the field site until transported on ice to the University of Pretoria. Extraction and analyses of fecal androgen metabolites were carried out according to Ganswindt *et al.*^60^ at the Endocrine Research Laboratory, University of Pretoria.

### Acoustic analyses

Acoustic data annotation was performed using a customized annotation tool from S_Tools Stx (Acoustic Institute, Austrian Academy of Science)^61^. Each rumble was identified based on field notes and by examining the spectrogram. The start and end cues of each rumble were tagged and the corresponding annotations were added.

Source-related acoustic features (fundamental frequency parameter) were analyzed using a customized semi-automatic analysis tool in Matlab^61^. The tool takes the segmented rumbles as input and computes a Fourier spectrogram using a frame size of 300 ms and a step size of 40 ms. Frequency contours are then traced within the spectrogram. From these contours, a number of features were extracted automatically. The features comprised a set of frequency-related parameters of the contour, shape and temporal structure (Table 2).

To analyze the filter-related formants, we downsampled the sound files to 2000 Hz and computed a LPC (Linear Predictive Coding)-smoothed spectrum in the range of 0 Hz to 500 Hz (LPC model order 16) using S_Tools Stx, and measured the center frequency of the LPC peaks/formants (although sometimes we could extract up to 4 formant peaks, only the first two peaks were consistently present in the vocalizations). In addition, we calculated formant dispersion of formant 1 and 2 (FΔ) and computed the estimated vocal tract lengths (VTL) for each vocalization using equation 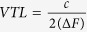. This equation assumes that the vocal tract is a uniform tube, and therefore anatomical and morphological deviations of the natural vocal tract from the uniform tube are not taken into account (therefore the term *estimated VTL* is used).

### Statistical analyses

The rumbles used for statistic analyses have been recorded in various different session and days over the data collection period (mean 6.4 ± 2.5, range 4 to 12). We used those rumbles that were recorded in low-affect social contexts. These included rumbles when elephants dispersed during feeding, rumbling while approaching another elephant or being approached, rumbling in reaction to physical contact, for example a trunk-touch (no agonistic interactions such as pushing or tusking), or during general locomotion of the group. We approximated balanced data sets and only considered males that contributed at least 10 rumbles (where all parameters could be analyzed) and randomly selected 20 rumbles of those individuals with more available recordings (Table 1). This resulted in 10 individuals that were used for statistical evaluation.

Since male elephants grow a lifetime, their size and age are generally highly correlated^44^. This was also true in our data set (Pearson Correlation, r = 0.942, p < 0.001). Therefore we combined age and size to the term ‘maturity’. Based on the available distribution, we divided the individuals into two maturity groups, (**1**) males with a shoulder height above 3 meter and older than 25 years (n_ind_ = 5), and group (**2**) with males younger than 25 years and a shoulder height below 3 meters (n_ind_ = 5).

We conducted pDFAs to test our ability to correctly classify rumbles to the maturity groups and individuals. The pDFA for nested design is a randomization procedure used for non-independent two-factorial data sets when one factor is nested in another (a test factor, a control factor and a restriction factor can be defined). The detailed procedure is described in Mundry and Sommer^62^. The pDFAs were conducted using a script written in software R (provided by R. Mundry). This script is based on the function Ida of the R package MASS^63^. The pDFA calculates the percentage of correctly classified objects for the original, unpermuted data, based on the calls used to derive discriminant functions and the percentage of correctly classified calls for the cross-validated (permuted) data, which were not used to derive discriminant functions^64^. For each pDFA, we used 100 random selections and 1000 permutations

Since the number of variables included in a DFA should be no more than the smallest number of cases at the level of the test factor^62^, we performed data reduction using principal component analysis (PCA). Underlying factors with eigenvalues above 1.0 were retained and varimax rotated. Factor scores were retained using the regression method and entered into the pDFAs instead of the original variables. The results are expressed as percentage of correct classification (cross-validated) and normalized against expected rates in term of error reduction (this term take into account the chance rate and, hence, produces an unbiased measure of the level of correct classification)^64^.

### Acoustic cues to maturity

In order to detect the most relevant discriminative variables, we first ran an ANOVA to test whether the mean values for each parameter differed significantly between maturity groups. These parameters were then entered into the PCA. The resulting factor scores were entered into the pDFA as variables, maturity group as test factor, and individuality as control factor.

### Acoustic cues to individuality

For the acoustic cues to individuality, we evaluated the Potential of Individual Coding (PIC)^65^,^66^, which calculates the ratio between within-individual variation (CV_w_) and between-individual variation (CV_b_) using the formula 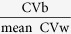, where mean CV_w_ is the mean value of the CV_w_ of all 10 individuals). Within-individual variation was calculated using the equation 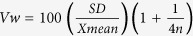, where *X*_mean_ is the mean of the sample and *n* is the sample size for one individual. CV_b_ was assessed according to the formula 
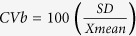
, where the standard deviation and *X*_*mean*_ are calculated for the entire sample. Acoustic parameters with a PIC > 1 have the potential to encode individual identity. These parameters were used for the PCA. The resulting factor scores were entered into the pDFA as variables, individuality as test factor, and maturity group as restriction factor (restricts the permutation to happen only within maturity groups).

All analyses were performed in SPSS v.22 (IBM Corp. released 2013. IBM SPSS Statistic for Macintosh, Version 22.0. Armonk, NY: IBM Corp.) and R version 3.2.4 (2016-03-10). Alpha values were set at 0.05.

## Additional Information

**How to cite this article**: Stoeger, A. S. and Baotic, A. Information content and acoustic structure of male African elephant social rumbles. *Sci. Rep.* **6**, 27585; doi: 10.1038/srep27585 (2016).

## Supplementary Material

Supplementary Information

## Figures and Tables

**Table 1:** Information on study subjects and data collection.

ID	Location/time period and duration (h) of recordings	Age^3^ (y)	*fAM (μg/g DW)* ± *SD*	Physical signs of *musth* (TGS+UD)^4^	App. Shoulder height (m)	Ncalls^5^
Mana	Pilanesberg /26.07.2014–6.08.2014/50h	∼29^1^	2.41±0.08	NO	∼3.25 m	13
Mike	Pilanesberg/see above	∼29^1^	2.27±0.74	NO	∼3.20 m	18
Sapi	Pilanesberg/see above	∼30^1^	2.33±0.74	NO	∼3.25 m	10
Sharu*	Pilanesberg/see above	∼30^1^		NO	∼3.20 m	2
Chichuru	Bela Bela/8.8.2014–13.8.2014/25h	∼18^2^	1.59±0.32	NO	∼2.40 m	20
Chova	Bela Bela/see above	∼21^2^	3.07	NO	∼2.50 m	20
Medwa	Hazyview/15.8.2014–30.8.2014/45h	∼19^2^	4.22	NO	∼2.60 m	15
Shamwari	Hazyview/see above	∼19^2^	5.35±0.23	NO	∼2.70 m	14
Tembo	Hazyview/see above	∼34^1^	5.37±1.32	NO	∼3.30 m	20
Ziziphus	Hazyview/see above	∼18^2^	5.65	NO	∼2.50 m	14
Duma	Addo/19.8.2015–26.8.2015/15h	∼28^1^	2.00	NO	∼3.25 m	18
Mukwa*	Addo/see above	∼29^1^	2.02	NO	∼3.25 m	5
Thaba*	Addo/see above	∼29^1^	2.08	NO	∼3.25 m	5

The ID, the location, the time and hours recorded at each institution, the age of the individuals, the signs of *musth*, the approximate shoulder height, and the number of calls entered into the data analysis.

^1^Maturity group 1: shoulder height >3 m and age >25 y;

^2^Maturity group 2: shoulder height <3 m and age <25 y.

^3^The exact date of birth is not known.

^4^The physical signs of *musth* are continued temporal gland secretion (TGS) and urine dribbling (UD)^21^.

^5^Number of calls entered into statistical analysis for individuality and age cues.

^*^Individuals’ not considered for statistical tests due to low sample size.

**Table 2:** Description of the acoustic parameters measured.

Acoustic parameter	Description
Absolute frequency parameter	
Start, mid, finish frequency	Fundamental frequency at the start, at the middle, and at the end of the rumble (Hz).
Min and max frequency	Lowest and highest measured frequency of the fundamental (Hz)
Mean frequency	Calculated as average frequency across the fundamental
Mean1^st^, 2^nd^ and 3^rd^ Third	mean fundamental frequencies of first, second, and third part of the sound segment (Hz)
Temporal parameters	
Duration	Temporal distance of rumble measured in seconds (s)
Min and max frequency loc	Location of the minimum and maximum frequency on the fundamental contour (s).
TimeMin/Max	Temporal distance between min and max frequency (s)
Shape and contour parameters	
COFM - Coefficient of frequency modulation	Calculated variable that represents the amount and magnitude of frequency modulation across a rumble, computed by summing the absolute values of the difference between sequential frequencies divided by 10,000.
Jitter Factor (Mitani and Brandt 1994)	Calculated variable that represents a weighted measure of the amount of frequency modulation, by calculating the sum of the absolute value of the difference between two sequential frequencies divided by the mean frequency. The sum result is then divided by the total number of points measured minus 1 and the final value is obtained by multiplying it by 100.
Frequency variability index (Mitani and Brandt 1994)	Calculated variable that represents the magnitude of frequency modulation across a rumble, computed by dividing the variance in frequency by the square of the average frequency of a rumble and then multiplying the value by 10.
Start, middle, and final slope	Calculated as (Frequency 20-Frequency 1)/(Time 20-Time 1) (Frequency 40-Frequency 20)/(Time 40-Time 20) (Frequency 60-Frequency 40)/(Time 60-Time 40)
Filter-related features	
Formant 1	First spectral peak of the LPC smoothed spectrum in the range of 0 to 500 Hz (model order 8)
Formant 2	Second spectral peak of the LPC smoothed spectrum in the range of 0 to 500 Hz (model order 8)

**Table 3:** Acoustic cues to maturity.

Variables	F_1,160_	Level of significance	Mean values ± SD	
			Maturity group 1	Maturity group 2
Formant 2	520.382	p < 0.001	77.14 Hz ± 7.6	104.53 Hz ± 7.7
Formant 1	343.144	p < 0.001	21.53 Hz ± 2.0	29.44 Hz ± 3.3
Max Frequency	133.041	p < 0.001	13.81 Hz ± 1.6	16.96 Hz ± 1.9
Min Frequency	130.844	p < 0.001	9.91 Hz ± 1.2	12.50 Hz ± 1.6
FinishFrequency	108.385	p < 0.001	10.41 Hz ± 1.5	13.20 Hz ± 1.9

Mean values ± SD of the most important acoustic parameters and significance levels of the ANOVA, comparing group means.
