# Supplementary material for: Information content and acoustic structure of male African elephant social rumbles
Source: Sci Rep. 2016 Jun 8;6:27585. doi: 10.1038/srep27585 (PMC4897791; doi:10.1038/srep27585)
Supplement: Supplementary Information [file srep27585-s1.pdf]

Supplemental Information

Information content and acoustic structure of male African elephant social rumbles

Angela. S. Stoeger[1,*] & Anton Baotic[1]

[1]Mammal Communication Lab, Department of Cognitive Biology, University of Vienna, Vienna, 1090, Austria

*angela.stoeger-horwath@univie.ac.at

Table S1. Principal Components Analysis Matrix for maturity groups, giving the percent variance explained and loading of variables for each principal component factor. The factor scores were entered into the pDFA for further analysis.

Matrix of components (rotated)

	Components			
	% Variance			
	50.7%	18.8%	7.3%	5.5%
	1	2	3	4
COFM	.268	.725	.074	.097
JitterFactor	-.026	.521	.650	.000
FinishFreq	.855	-.165	.181	.204
MinFreq	.808	-.288	.230	.015
MaxFreq	.907	.332	.199	.112
MeanFreq	.972	.120	.168	.085
FreqRange	.258	.924	.017	.162
PeakByMeanFreq	-.090	.814	.110	.075
MeanByMinFreq	-.110	.821	-.249	.121
PeakFreqLoc	.090	.101	.105	.876
Duration	.090	.353	.048	.494
StartFreq	.772	-.164	.225	-.348
MidFreq	.884	.264	.121	.124
mean1stThird	.923	.053	.157	-.194
mean2ndThird	.713	.259	.135	.127
mean3rdThird	.793	.031	.186	.279
medianFreq	.905	.144	.141	.087
Formant1	.801	.268	.543	.243
Formant2	.923	-.122	.785	.116
F2-F1	.452	-.249	.755	.051

Table S2. **Matrix of components of the PCA for analyzing acoustic cues to individuality.** giving the % percent variance explained and loading of variables for each principal component factor. The factor scores were entered into the pDFA for further analysis.

Matrix of components (rotated)

	Components					
	% Variance					
	42.5%	14.6%	7.6%	6.2%	5.5%	5.3%
	1	2	3	4	5	6
COFM	.260	.811	-.019	.298	.060	.027
JitterFactor	-.004	.518	.573	.035	.113	-.181
InflectionFactor	.041	.014	.110	.742	-.085	-.306
FinishFreq	.859	-.095	.125	.267	.159	.212
MinFreq	.916	-.254	.195	.168	.032	.066
MaxFreq	.913	.329	.164	-.036	.075	.034
MeanFreq	.971	.149	.157	-.020	.038	.055
FreqRange	.259	.874	.006	-.282	.079	-.033
MeanByMinFreq	-.131	.794	-.188	-.417	-.007	-.015
PeakFreqLoc	.105	.183	.117	-.019	.745	.249
Duration	.066	.470	.119	-.007	.267	.120
StartSlope	.059	.091	-.013	-.320	-.337	.724
MiddleSlope	.014	.109	-.016	-.097	.871	-.229
FinalSlope	-.044	-.175	-.049	.749	-.035	.294
StartFreq	.796	-.220	.166	.098	-.152	-.397
MidFreq	.882	.252	.129	-.180	.071	.101
TimeMinMax	-.123	.024	-.053	-.165	-.171	-.601
mean1stThird	.923	.049	.154	-.042	-.230	-.105
mean2ndThird	.911	.253	.144	-.191	.094	.087
mean3rdThird	.892	.118	.151	.183	.216	.163
medianFreq	.950	.177	.145	-.063	.030	.053
Formant1	.495	.359	.561	.045	.077	.131
Formant2	.532	-.076	.806	.041	.041	.074
F2-F1	.464	-.227	.775	.034	.021	.042

Figures

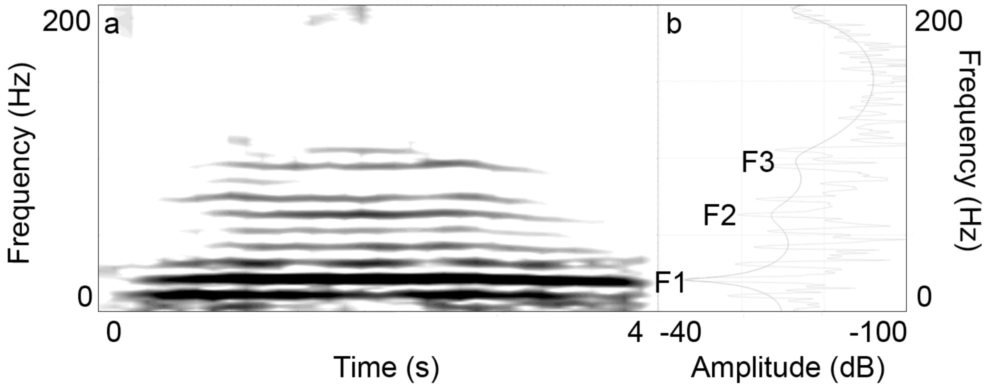

Figure S1. Spectrogram (a) and spectrum (b) of a rumble produced by Mike (age ~ 29 years, shoulder height ~3.20 m). Three formants are measurable: F1 = 22 Hz, F2 = 64 Hz, F3 = 100 Hz.

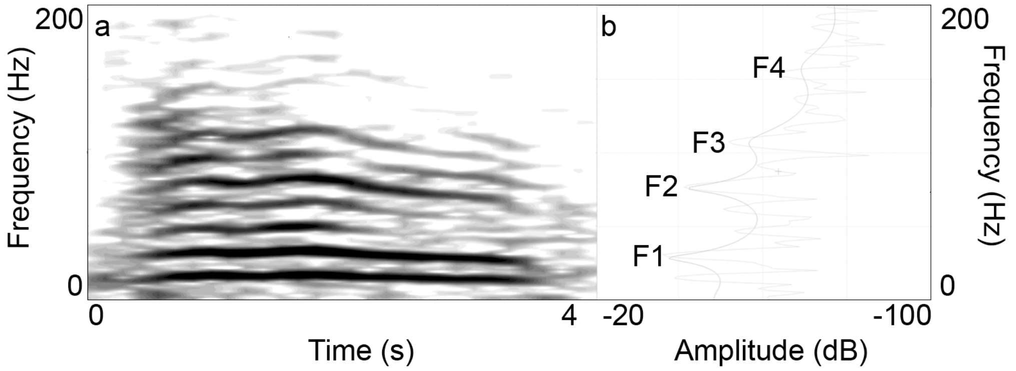

Figure S2. Spectrogram (a) and spectrum (b) of a rumble produced by Tembo (age ~ 34 years, shoulder height ~3.40 m). Four formants are measurable: F1 = 26 Hz, F2 = 74 Hz, F3 = 114 Hz, F4 = 170 Hz.

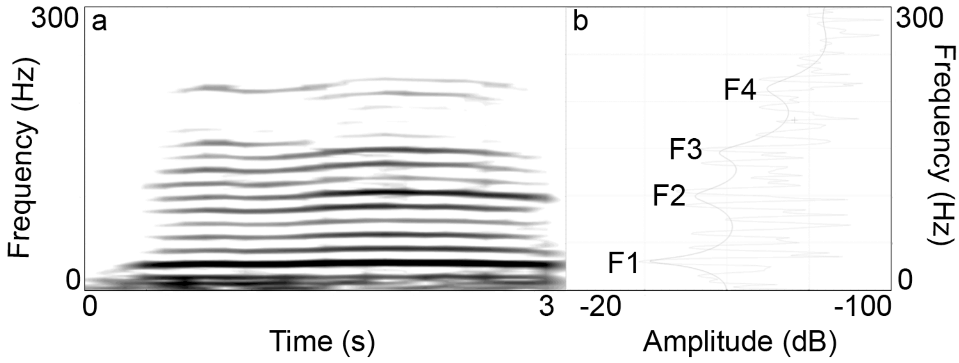

Figure S3: Spectrogram (a) and spectrum (b) of a rumble produced by Chova (age ~ 21 years, shoulder height ~2.50 m). Four formants are measurable: F1 = 28 Hz, F2 = 100 Hz, F3 = 145 Hz, F4 = 207 Hz.
